# Optimized Loading of Carboxymethyl Cellulose (CMC) in Tri-component Electrospun Nanofibers Having Uniform Morphology

**DOI:** 10.3390/polym12112524

**Published:** 2020-10-29

**Authors:** Motahira Hashmi, Sana Ullah, Azeem Ullah, Muhammad Akmal, Yusuke Saito, Nadir Hussain, Xuehong Ren, Ick Soo Kim

**Affiliations:** 1 Nano Fusion Technology Research Group, Shinshu University Ueda Campus, Nagano 386-8567, Japan; motahirashah31@gmail.com (M.H.); sanamalik269@gmail.com (S.U.); 08tex101@gmail.com (A.U.); yusukesaito1458@gmail.com (Y.S.); engr.nadir712@hotmail.com (N.H.); 2 Department of Polymer Engineering, National Textile University, Faisalabad, Punjab 37610, Pakistan; akmalmalik375@gmail.com; 3 Key Laboratory of Eco-Textiles of Ministry of Education, College of Textiles Science & Engineering, Jiangnan University, Wuxi 214122, China; xhren@jiangnan.edu.cn

**Keywords:** carboxymethyl cellulose, electrospinning, polyvinyl alcohol, polyvinylpyrrolidone, uniform morphology

## Abstract

Cellulose is one of the most hydrophilic polymers with sufficient water holding capacity but it is unstable in aqueous conditions and it swells. Cellulose itself is not suitable for electrospun nanofibers’ formation due to high swelling, viscosity, and lower conductivity. Carboxymethyl cellulose (CMC) is also super hydrophilic polymer, however it has the same trend for nanofibers formation as that of cellulose. Due to the above-stated reasons, applications of CMC are quite limited in nanotechnology. In recent research, loading of CMC was optimized for electrospun tri-component polyvinyl alcohol (PVA), polyvinylpyrrolidone (PVP), and carboxymethyl cellulose (CMC) nanofibers aim at widening its area of applications. PVA is a water-soluble polymer with a wide range of applications in water filtration, biomedical, and environmental engineering, and with the advantage of easy process ability. However, it was observed that only PVA was not sufficient to produce PVA/CMC nanofibers via electrospinning. To increase spinnability of PVA/CMC nanofibers, PVP was selected as the best available option because of its higher conductivity and water solubility. Weight ratios of CMC and PVP were optimized to produce uniform nanofibers with continuous production as well. It was observed that at a weight ratio of PVP 12 and CMC 3 was at the highest possible loading to produce smooth nanofibers.

## 1. Introduction

Advancement in technologies, product, and system design have brought revolution in the lifestyle of mankind. However, continuous development is key to sustaining a developing society. Nanotechnology is one of the most advanced technologies, which covers a wide range of applications [1,2,3]. Electrospinning is a technique to produce the nonwoven mats that offer a large surface-area-to-mass ratio. Nanofibers produced by electrospinning have a diameter in the range of some nanometers to sub-micron while the length of nanofibers can be in the range of some microns to sub-millimeter [4,5,6,7,8,9,10]. Due to high surface area, nanofibers from hydrophilic polymers may have the best utilization in water adsorption or absorption. However, applications of electrospun nanofibers are not limited to water treatment/adsorption only, but cover tissue engineering, biomedical engineering, energy storage, sensors and actuator, food packaging, air filtration, antibacterial, and antiviral nanofibrous products as well [11,12,13,14,15,16,17]. Hydrogels are very good in their water absorption properties. They have three dimensional macromolecular networks, which swell in the presence of water but do not dissolve in the water. They possess excellent water absorption due to the hydrophilic functional groups, which are attached to their backbone chain [18,19].

Natural, semi-synthetic, and synthetic polymers have a wide range of applications in materials science and engineering. However, sustainable development is a key consideration for modern research. Cellulose is one of the most useful natural polymers having number of applications in tissue engineering, water treatment, filters, food and packaging industry, and other areas of science as well [20,21,22,23,24]. However, applications of cellulose are limited in the nanofibers-based products due to high swelling, gel formation, and processing difficulties. To widen the area of applications for cellulose, it is modified into different semi-synthetics like cellulose acetate, CMC, hydroxyethyl cellulose, and other sub types of cellulose [25,26,27,28]. Carboxymethyl cellulose CMC is famous due to its versatile applications in different fields like drug delivery, tissue engineering, food industry, cosmetics, printing, and dying. CMC also gains attention due to its water holding capacity. It possesses good water holding capacity even at low temperature. CMC has good water absorption properties in all forms, but the absorption rate is higher in film than the fiber. It is reported that 6000% of water has been absorbed by the CMC film from its initial mass while in fiber form, it is 2000%. The water-absorbing property of CMC is due to the presence of hydroxyl groups. Hydroxyl groups work as the bonding sites for the water through hydrogen bonding [29,30]. CMC is hydrophilic polymer. It can be easily dissolved in cold water without gel formation. It does not form a gel in cold water because unsubstituted sites of the backbone chain are not fully active and do not work. Meanwhile, the gel formation rate increases as the temperature of water increase because of unsubstituted sites of cellulose along the backbone chain act as temporary cross linkers between the chains. Viscosity of the CMC increase due to the gel formation and it became very difficult to electrospun.

PVA is a polymer with the decreasing trend of viscosity as the temperature increase. The presence of hydroxyl groups on PVA create inter and intra molecular hydrogen bonding. Hydrogen bonding is the reason for the strong interaction between CMC and PVA. The mechanical properties of the blend are also very good. The blend possesses smooth morphology with no bead formation. However, there is bead formation as the concentration of CMC increase above 5%. The absorption rate of CMC may decrease by adding the PVA. To maintain the good absorption level, it is necessary to introduce the strong hydrophilic polymer to the blend [31,32,33]. PVP is a very good hydrophilic polymer with good biocompatibility, excellent film-forming properties, non-toxicity, biodegradability, and low surface tension [34,35]. PVP also possess electrical conductivity as its intrinsic characteristic and charge storage capacity [36,37]. PVP has a strong interaction with both CMC and PVA. Carbonyl groups are present on the backbone chain of the PVP, which make inter-chain hydrogen bond with the hydroxyl groups present on the PVA chain.

In this research, PVP and PVA were selected as carriers for CMC. The main objective of this research was to obtain smooth nanofibers with continuous electrospinning. Addition of CMC can enhance some of the characteristic properties of nanofibers, which further widened the area of applications for PVA and PVP. CMC’s excellent water holding capacity, biocompatibility, biodegradability, and other characteristics can be used in a number of applications such as food packaging (nanofibers based), biomedical, and environmental engineering applications. This is a novel idea to load maximum possible amount of CMC on electrospinning to get uniform nanofibers that can be utilized in practical applications. In future, authors have plans to carry on this idea to further characterize tri-component nanofibers consisting of optimized ratios of PVA, PVP, and CMC for mechanical, biodegradability, and water holding capacity to specific application areas for the betterment of society.

## 2. Materials and Methods

Polyvinylpyrrolidone (PVP) with an average molecular weight of 40,000 was purchased from Sigma-Aldrich (St. Louis, MO, USA), Polyvinyl alcohol (PVA) with an average molecular of 85,000–124,000 and 87–89% hydrolyzed was purchased from Sigma-Aldrich, and sodium carboxymethyl cellulose (Na-CMC) with an average molecular weight of 250,000 and degree of substitution of 1.2 was also purchased from Sigma-Aldrich chemicals. All materials were used as received without chemical or physical modification.

Concentration of PVA in spinning solution was kept constant (6% *w*/*w*) while weight percentage of PVP and CMC were varied. PVP was added from 10 weight percent to 14 weight percent while CMC was added at the lowest from 1 percent to 3 weight percent as shown in Table 1.Distilled water was used as solvent for PVA, CMC, and PVP. Stated quantities of polymers were blended and stirred for 8 h at 60 °C to get homogenous solution. Viscosity of each spinning solution (3 samples for each solution) was measured by a viscometer using 63-number cylinder. Electrospinning was carried out at a voltage of 20 kV, the distance from tip to collector (cooking sheet was wrapped on collecting drum to get nanofibrous mats) was kept at 18 cm, and the flow rate of spinning solution was set to 1.0 mL/h. All samples were prepared following the same procedure and conditions. After electrospinning, all each sample was kept in airtight plastic bag at room temperature for further characterization.

### Characterization

To investigate the chemical reaction between CMC, PVP, and PVA, FTIR with an ATR prestige-21 (Shimadzu, Japan) was used. Fingerprints of ATR were recorded from 600 to 4000 cm^−1^. Scanning electron microscope (SEM, JSM-5300, JEOL Ltd., Akishima, Japan) was used to check the surface properties of nanofiber mats at the voltage of 10 kV. Average diameter was calculated by image analysis software (Image J, version 1.4.3) from 50 readings of random nanofibers of each of the samples. Water contact angle was investigated by using contact angle analyzer (Digidrop, GBX, Bourg-de-Peage, France). Thermal analysis of PVA/CMC, PVA/PVP, and PVA/PVP/CMC composite nanofibers was examined by thermogravimetric analyzer Thermo-plus TG 8120 (Rigaku Corporation, Osaka, Japan). TGA test was performed under ambient (air) atmosphere in static mode and heating rate was set to 10 °C/min and 25–500 °C temperature range for all specimen. Water contact angle (WCA) was measured using contact angle analyzer (Digidrop, GBX). The volume of water droplet was set to 2 µL while pictures were captured after 1 s (1000 milli seconds).

## 3. Results and Discussions

### 3.1. Fourier Transform Infrared Spectroscopy (FTIR)

**Figure 1:**
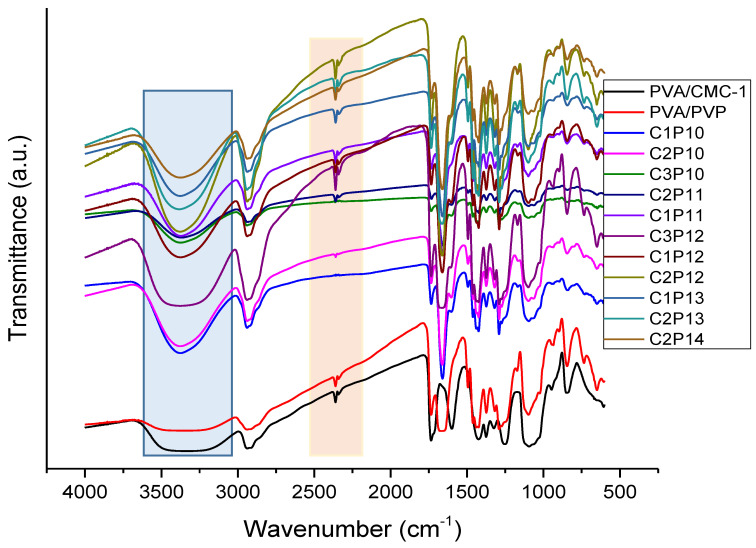
FTIR-ATR spectra of polyvinyl alcohol (PVA)/polyvinylpyrrolidone (PVP)/carboxymethyl cellulose (CMC) composite nanofibers with varying weight ratios of PVP and CMC.

All three polymers used in this study are highly hydrophilic in nature, and the reason behind hydrophilicity is the presence of abundant hydroxyl groups in main chains of polymeric structures. FTIR-ATR spectra in Figure 1 show the presence of hydroxyl groups (–OH peaks) found in the spectra of PVA, PVA/PVP, PVA/CMC, and PVA/PVP/CMC nanofiber mats. In the case of PVA/CMC–1 and PVA/PVP, the hydroxyl peak (–OH) was found to be broader as compared to that of composite nanofibers containing all three polymers. In Figure 1, a broader peak was observed at the wavenumber of 3150–3450 cm^−1^, which was associated with the presence of hydroxyl groups in PVA and PVP main chains, however the same peak became sharper when the concentration of PVP and CMC was increased in composite nanofibers, i.e., a sharp peak was observed at 3402 cm^−1,^ which is generic peak of the hydroxyl group. Further, in the results of the water contact angle, it was also confirmed that increasing percentage of CMC in composite nanofibers imparted super-hydrophilicity to the nanofibrous mats. Characteristic peaks of PVA were observed at 3200 to 3500 cm^−1^, which indicates the presence of hydroxyl group (–OH stretching) in PVA chains. A peak at 2900 cm^−1^ was also observed, which referred to (–CH_2_–) asymmetric and symmetric band [19]. In the case of PVP, the peak around 1650 cm^−1^ can be associated with stretching vibration of the C=O in the pyrrolidone group, while CH stretching can be assigned to the absorption peaks around 2850 to 2980 cm^−1^. CH deformation bands can be associated to bands at 1430 and 1370 cm^−1^ (difference in peaks in red and black in given spectra). C–N bending vibration of pyrrolidone can be associated with the band at 1279 cm^−1^, however PVP did not show any significance peak at 3400–3500 cm^−1^, which are usually associated with the presence of amines [38]. It is highly expected that PVA, PVP, and CMC could form hydrogen bonding among their chains as it helped in uniform processing and homogenous mixing of the tri-component blend of three different polymers.

### 3.2. Morphological Properties

**Figure 2:**
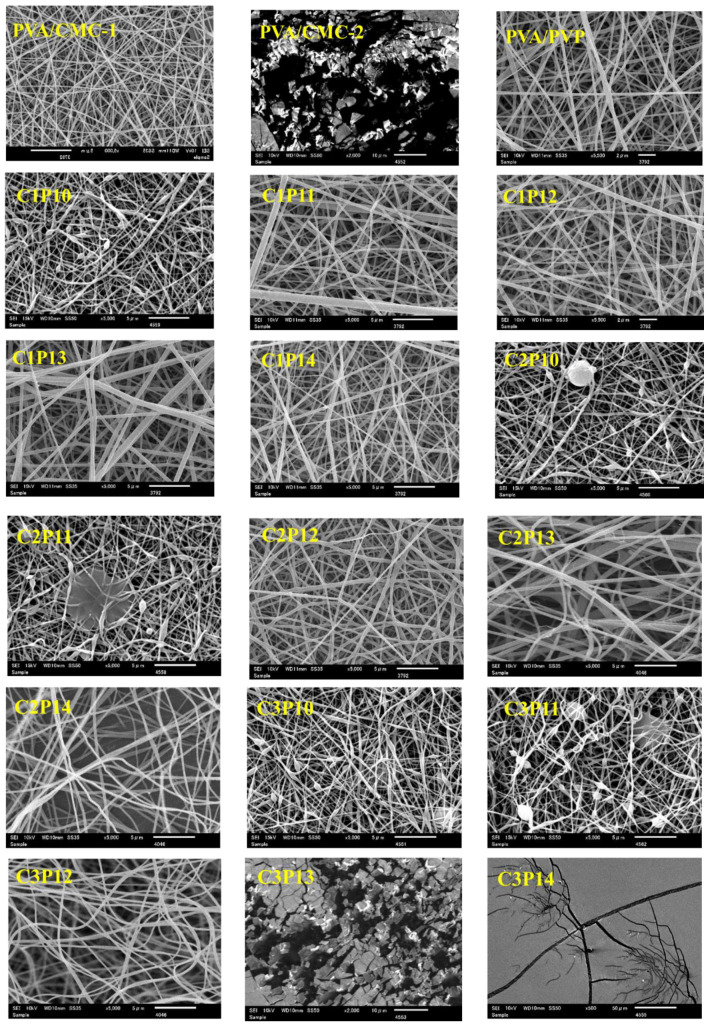
SEM images of PVA/CMC (PVA/CMC 1–2), PVA/PVP, and PVA/PVP/CMC (C1P10–C3P14) composite nanofibers: Effect of variation of concentration of PVP and CMC on morphology of nanofibrous mats.

Morphological properties like surface structure and nanofibers’ diameter were observed by the scanning electron microscope (SEM). As it was also stated in the introduction section, CMC cannot be electrospun due to high viscosity and low conductivity. However, PVA is easily electrospun even at varying concentrations (6% to 10% is most suitable for smoother nanofibers). PVP is also very difficult to electrospin at lower concentrations due to lower viscosity. The addition of PVP lowers the viscosity of CMC solution as well as imparts conductivity to the spinning solution and makes it electrospinnable with good nanofiber formation. Figure 2 represents SEM images of PVA/PVP, PVA/CMC, and PVA/PVP/CMC composite nanofibers with varying concentrations of CMC (1-3 weight ratio) and PVP (10–14 weight ratio). In Figure 2 it can be observed that PVA/CMC-1 (PVA:CMC = 6:1) exhibited smoother nanofibers while not a single nanofiber was observed by increasing CMC concentration to 6:2 (*w*/*w*). PVA/PVP nanofibers were also found to be smoother and finer. The addition of PVP in spinning solution provided support of conductivity and viscosity, which resulted smoother nanofibers in case of C1P10, C1P11, C1P12, and even C1P14. However, the addition of further CMC (weight ratio of 2) did not show the same trend as that of CMC1. Samples C2P10 and C2P11 nanofibers were not uniform and have some beads on the surface, while C2P13 and C2P14 nanofibers had uniform morphology. The ratio of CMC was further increased to a weight ratio of 3 to confirm the maximum loading capacity of CMC for smoother nanofibers. It was observed that for CMC-3, only the C3P12 sample exhibited smoother morphology while C3P10 and C3P11 samples had beads on the nanofibers’ surface; on the other hand, not a single nanofiber was observed in the case of C3P13 and C3P14. In conclusion of morphological characterization, it can be stated that maximum and optimum loading capacity for CMC is up to a weight ratio of 3 with respect to PVA and PVP, while the most suitable concentration for smoother and continuous nanofibers production without beads formation is PVA:PVP:CMC = 6:12:3.

### 3.3. Diameter Distribution of Nanofibers

**Figure 3:**
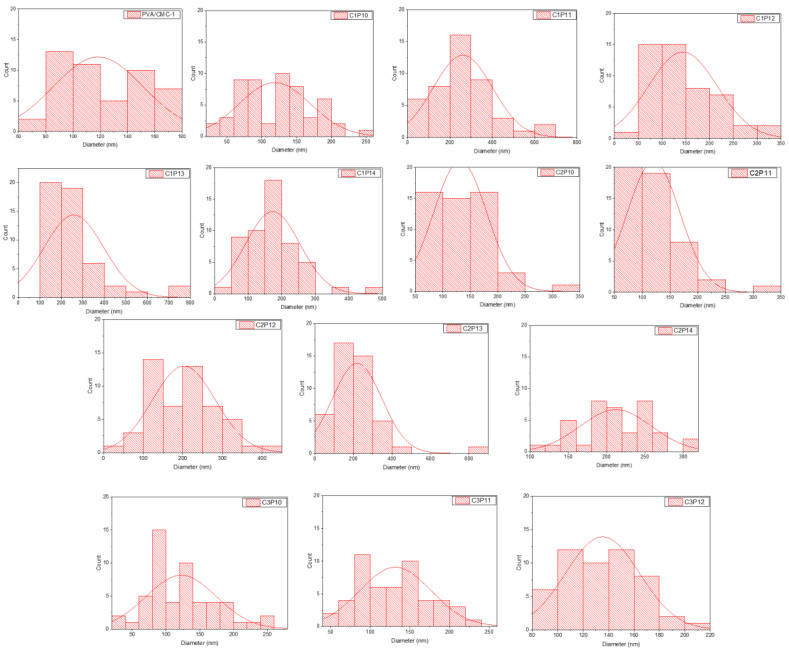
Diameter distribution plots (histogram) of PVA/CMC, PVA/PVP, and tri-component composite nanofibers of PVA, PVP, and CMC (C1P10–C3P12).

Diameters of nanofibers were measured by Image J. software by taking 50 random readings of nanofibers for each sample. Figure 3 represents diameter distribution trend as shown in histograms of each sample (here samples represent only polymer compositions that were easily converted to nanofibers on electrospinning). It can be shown that PVA/CMC-1 samples exhibited uniform nanofibers of diameter range 80 to 180 nm having an average diameter of 120 nm, which indicates successful conversion of polymers in to nanofibers. However, PVA/CMC-2 (having PVA:CMC = 6:2) did not show any sign of nanofiber formation. The addition of further CMC and PVP in PVA solution caused some diversity in the diameter distribution of nanofibers. However, it was observed that samples containing 12% PVP exhibited nanofibers of finer and a uniform diameter range. It may be because of compatibility or the formation of bonding among three polymers on this specific composition. However, this claim needs further confirmation after performing the relevant test. As it can be observed in Figure 2 and Figure 3, that with 12% PVP and varying concentration of CMC exhibited uniform nanofibers without beads formation, as well as uniform diameter distribution.

### 3.4. Water Contact Angle (WCA)

**Figure 4:**
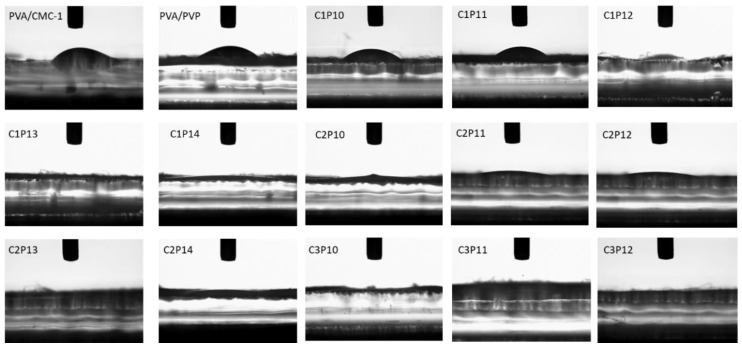
Water contact angles (WCA) for PVA/CMC, PVA/PVP, and PVA/PVP/CMC (C1P10 to C3P12) nanofibers: Effect of variation in weight ratios of PVP and CMC on water affinity of nanofibrous mats.

The water-holding capacity of any substance is generally dependent on hydrophilic or hydrophobic nature [18]. Hydrophilicity can be assessed by measuring water contact angle (WCA). WCA of PVA/CMC, PVA/PVP/CMC, and PVA/PVP was measured to evaluate hydrophilic capacity of electrospun nanofibers. Uncrosslinked PVA nanofibers generally have hydrophilic nature while crosslinked nanofibers exhibit hydrophobicity depending upon the degree and type of crosslinking [19]. CMC and PVP are also highly hydrophilic polymers. The selection of hydrophilic polymers for a Tricomponent blend for enhanced adsorption and absorption properties was also one of the objectives of this research (however further testing will be carried out in future research regarding adsorption and absorption characteristics to evaluate usability of prepared blends for such applications commercially). Figure 4 represents WCA for composite nanofibrous mats. It can be seen that water contact angles for all of the nanofibrous mats are in the range of hydrophilic, however samples a-d exhibited contact angle ranging from approximately 15° to 20° while WCA values for samples e-o was found to be at 0° (except samples I and J, but average for these two samples were also well below 1°). It was observed that increasing PVP content did not bring any significant changes in water contact angles of composite nanofibers while increasing CMC content significantly decreased WCA values for composite nanofibers, which indicates that CMC has more tendency towards water due to containing abundant hydroxyl groups in main chain as compared to that of PVA or PVP. The addition of optimum content of CMC will impart hydrophilicity to the composites. It is suggested that CMC should be added with hydrophobic polymers to increase their tendency towards hydrophilic nature. However, compatibility of polymers should be properly examined before blending with CMC.

### 3.5. Thermogravimetric Analysis (TGA)

**Figure 5:**
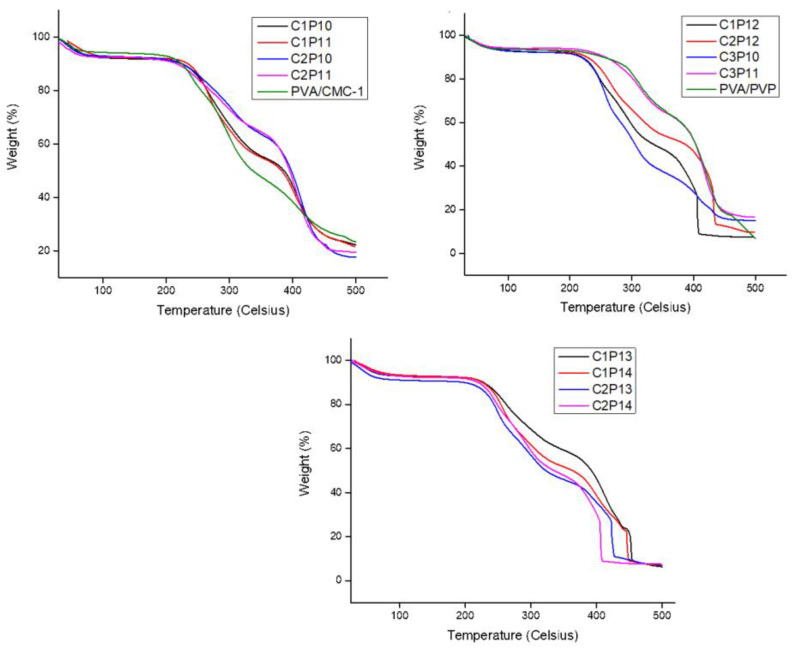
Thermogravimetric analysis (TGA) plot of PVA/PVP, PVA/CMC, and tri-component PVA/PVP/CMC (C1P10 to C3P12) nanofibers: Effect of variation in weight ratios of PVP and CMC on thermal properties of nanofibrous mats.

CMC is generally unstable above 280 °C, while PVA and PVP are thermally stable well above 300 °C. Figure 5 represents TGA curves of PVA/CMC, PVA/PVP, and PVA/PVP/CMC (with varying ratios of PVP and CMC). TGA plots in Figure 5 have been divided in three groups due to the overlapping of results. Generally, a TGA curve is divided in three parts on the basis of temperature zones [39]; the first temperature zone is up to 100 °C, which indicates evaporation of high volatile components including impurities and vapors. The second zone starts from onset temperature and ends at offset temperature of substance. The second zone describes the thermal stability of substance, while the third and the last temperature zone starts from offset temperature of substance, which shows burning or degradation of substance. For substances/polymers that are not thermally stable, the last zone is usually flatter as compared to that of being thermally stable. It can be observed that the onset temperatures for PVA/PVP nanofibrous mats was well above 300 °C while the onset temperature of nanofibers containing CMC was dropped to 220–250 °C, which indicated that addition of CMC in tri-component nanofibrous mats caused decrement in thermal stability of polymers. However, considering practical applications such as food packaging and water absorption/adsorption, the thermal stability of nanofibers is still enough to be used as it is. It was also observed that at lower weight ratios of CMC and PVP the third onset was not so clearly visible while increasing content of PVP and CMC, and the third onset (curve) is clearly visible in TGA plot. Increasing CMC content also caused a decrease in residue.

## 4. Conclusions

Considering the results and discussions of experimental section, it can be concluded that CMC is not suitable for electrospinning as a single component, however it can be processed by forming blends/composites with compatible polymers like PVA and PVP. It was observed that specific concentrations of CMC and PVP formed highly uniformed nanofibers without any bead formation. Processability was also observed to be smoother specifically for samples containing 12 wt.% PVP. There was no significance on thermal degradation due to addition of CMC. Water contact angle was further decreased to 0° with the addition of CMC in tri-component nanofibers. Nanofibers with smoother morphology will have potential applications in the field of biomedical, agriculture, and environmental engineering.

## Figures and Tables

**Table 1:** Ratio of polymers (*w*/*w*) in tri-component composite nanofibrous mats and viscosities of spinning solutions with standard deviation.

Sample Code	Polymers’ Composition			Viscosity	Remarks
	PVA	PVP	CMC	cps	
PVA/CMC-1	6	0	1	181 ± 7	Nanofiber formation
PVA/CMC-2	6	0	2	238 ± 5	No nanofiber observed
PVA/PVP	6	12	0	157 ± 4	Nanofiber formation
C1P10	6	10	1	141 ± 5	Nanofiber formation
C1P11	6	11	1	161 ± 4	Nanofiber formation
C1P12	6	12	1	187 ± 6	Nanofiber formation
C1P13	6	13	1	201 ± 7	Nanofiber formation
C1P14	6	14	1	218 ± 3	Nanofiber formation
C2P10	6	10	2	197 ± 5	Nanofiber formation
C2P11	6	11	2	204 ± 5	Nanofiber formation
C2P12	6	12	2	215 ± 4	Nanofiber formation
C2P13	6	13	2	237 ± 6	Nanofiber formation
C2P14	6	14	2	249 ± 8	Nanofiber formation
C3P10	6	10	3	236 ± 6	Nanofiber formation
C3P11	6	11	3	247 ± 4	Nanofiber formation
C3P12	6	12	3	252 ± 3	Nanofiber formation
C3P13	6	13	3	277 ± 5	No nanofiber observed
C3P14	6	14	3	286 ± 6	No nanofiber observed

## References

[1] Ullah S., Ullah A., Lee J., Jeong Y., Hashmi M., Zhu C., Joo K.I., Cha H.J., Kim I.S. (2020). Reusability Comparison of Melt-Blown vs Nanofiber Face Mask Filters for Use in the Coronavirus Pandemic. ACS Appl. Nano Mater..

[2] Mayakrishnan G., Somasundaram S., Ullah S., Andivelu I., Soo K.I., Ill-Min C.I. (2019). Facile Green Preparation of Rhodium Nanoclusters Supported Nano-Scaled Graphene Platelets for Sonogashira Coupling Reaction and Reduction of p-Nitrophenol. Catalysts.

[3] Gopiraman M., Saravanamoorthy S., Ullah S., Ilangovan A., Kim I.S., Chung I.M. (2020). Reducing-agent-free facile preparation of Rh-nanoparticles uniformly anchored on onion-like fullerene for catalytic applications. RSC Adv..

[4] Ullah S., Hashmi M., Kharaghani D., Khan M.Q., Saito Y., Yamamoto T., Lee J., Kim I.S. (2019). Antibacterial properties of in situ and surface functionalized impregnation of silver sulfadiazine in polyacrylonitrile nanofiber mats. Int. J. Nanomed..

[5] Hashmi M., Ullah S., Kim I.S. (2019). Copper oxide (CuO) loaded polyacrylonitrile (PAN) nanofiber membranes for antimicrobial breath mask applications. Curr. Res. Biotechnol..

[6] Ullah S., Hashmi M., Khan M.Q., Kharaghani D., Saito Y., Yamamoto T., Kim I.S. (2019). Silver sulfadiazine loaded zein nanofiber mats as a novel wound dressing. RSC Adv..

[7] Hussain N., Yousif M., Ali A., Mehdi M., Ullah S., Ullah A., Mahar F.K., Kim I.S. (2020). A facile approach to synthesize highly conductive electrospun aramid nanofibers via electroless deposition. Mater. Chem. Phys..

[8] Bie X., Khan M.Q., Ullah A., Malik S., Kharaghani D., Duy P.N., Tamada Y., Kim I.S. (2020). Fabrication and characterization of wound dressings containing gentamicin/silver for wounds in diabetes mellitus patients. Mater. Res. Express.

[9] Khan M.Q., Kharaghani D., Nishat N., Sanaullah, Shahzad A., Hussain T., Kim K.O., Kim I.S. (2019). The fabrications and characterizations of antibacterial PVA/Cu nanofibers composite membranes by synthesis of Cu nanoparticles from solution reduction, nanofibers reduction and immersion methods. Mater. Res. Express.

[10] Kharaghani D., Suzuki Y., Gitigard P., Ullah S., Kim I.S. (2020). Development and characterization of composite carbon nanofibers surface-coated with ZnO/Ag nanoparticle arrays for ammonia sensor application. Mater. Today Commun..

[11] Kharaghani D., Gitigard P., Ohtani H., Kim K.O., Ullah S., Saito Y., Khan M.Q., Kim I.S. (2019). Design and characterization of dual drug delivery based on in-situ assembled PVA/PAN core-shell nanofibers for wound dressing application. Sci. Rep..

[12] Hashmi M., Ullah S., Kim I.S. (2020). Electrospun Momordica Charantia Incorporated Polyvinyl Alcohol (PVA) Nanofibers for Antibacterial Applications. Mater. Today Commun..

[13] Khan M.Q., Kharaghani D., Sanaullah, Shahzad A., Saito Y., Yamamoto T., Ogasawara H., Kim I.S. (2019). Fabrication of antibacterial electrospun cellulose acetate/silver-sulfadiazine nanofibers composites for wound dressings applications. Polym. Test.

[14] Khan M.Q., Kharaghani D., Sanaullah, Shahzad A., Ha Y., Duysegawa N.P., Azeemullah, Lee J., Kim I.S. (2020). Fabrication of Antibacterial Nanofibers Composites by Functionalizing the Surface of Cellulose Acetate Nanofibers. ChemistrySelect.

[15] Kharaghani D., Khan M.Q., Tamada Y., Ogasawara H., Inoue Y., Saito Y., Hashmi M., Kim I.S. (2018). Fabrication of electrospun antibacterial PVA/Cs nanofibers loaded with CuNPs and AgNPs by an in-situ method. Polym. Test.

[16] Ullah A., Ullah S., Khan M.Q., Hashmi M., Nam P.D., Kato Y., Tamada Y., Kim I.S. (2020). Manuka honey incorporated cellulose acetate nanofibrous mats: Fabrication and in vitro evaluation as a potential wound dressing. Int. J. Biol. Macromol..

[17] Khan M., Kharaghani D., Ullah S., Waqas M., Abbasi A., Saito Y., Zhu C., Kim I. (2018). Self-Cleaning Properties of Electrospun PVA/TiO_2_ and PVA/ZnO Nanofibers Composites. Nanomaterials.

[18] Hashmi M., Ullah S., Ullah A., Khan M.Q., Hussain N., Khatri M., Bie X., Lee J., Kim I.S. (2020). An optimistic approach “from hydrophobic to super hydrophilic nanofibers” for enhanced absorption properties. Polym. Test.

[19] Ullah S., Hashmi M., Hussain N., Ullah A., Sarwar M.N., Saito Y., Kim S.H., Kim I.S. (2020). Stabilized nanofibers of polyvinyl alcohol (PVA) crosslinked by unique method for efficient removal of heavy metal ions. J. Water Process Eng..

[20] Yang X., Biswas S.K., Han J., Tanpichai S., Li M.C., Chen C., Zhu S., Das A.K., Yano H. (2020). Surface and Interface Engineering for Nanocellulosic Advanced Materials. Adv. Mater..

[21] Han J., Wang S., Zhu S., Huang C., Yue Y., Mei C., Xu X., Xia C. (2019). Electrospun Core-Shell Nanofibrous Membranes with Nanocellulose-Stabilized Carbon Nanotubes for Use as High-Performance Flexible Supercapacitor Electrodes with Enhanced Water Resistance, Thermal Stability, and Mechanical Toughness. ACS Appl. Mater. Interfaces.

[22] Ding Q., Xu X., Yue Y., Mei C., Huang C., Jiang S., Wu Q., Han J. (2018). Nanocellulose-Mediated Electroconductive Self-Healing Hydrogels with High Strength, Plasticity, Viscoelasticity, Stretchability, and Biocompatibility toward Multifunctional Applications. ACS Appl. Mater. Interfaces.

[23] Zheng C., Lu K., Lu Y., Zhu S., Yue Y., Xu X., Mei C., Xiao H., Wu Q., Han J. (2020). A stretchable, self-healing conductive hydrogels based on nanocellulose supported graphene towards wearable monitoring of human motion. Carbohydr. Polym..

[24] Han J., Wang H., Yue Y., Mei C., Chen J., Huang C., Wu Q., Xu X. (2019). A self-healable and highly flexible supercapacitor integrated by dynamically cross-linked electro-conductive hydrogels based on nanocellulose-templated carbon nanotubes embedded in a viscoelastic polymer network. Carbon N. Y..

[25] Ci J., Cao C., Kuga S., Shen J., Wu M., Huang Y. (2017). Improved Performance of Microbial Fuel Cell Using Esterified Corncob Cellulose Nanofibers to Fabricate Air-Cathode Gas Diffusion Layer. ACS Sustain. Chem. Eng..

[26] Song J., Chen C., Yang Z., Kuang Y., Li T., Li Y., Huang H., Kierzewski I., Liu B., He S. (2018). Highly Compressible, Anisotropic Aerogel with Aligned Cellulose Nanofibers. ACS Nano.

[27] Dizge N., Shaulsky E., Karanikola V. (2019). Electrospun cellulose nanofibers for superhydrophobic and oleophobic membranes. J. Memb. Sci..

[28] Zhu M., Wang Y., Zhu S., Xu L., Jia C., Dai J., Song J., Yao Y., Wang Y., Li Y. (2017). Anisotropic, Transparent Films with Aligned Cellulose Nanofibers. Adv. Mater..

[29] Boruvková K., Wiener J., Jakubičková M. (2012). Preparation and properties of microporous structures based on cmc. Proceedings of the NANOCON 2012 4th International Conference.

[30] Tajeddin B., Ramedani N. (2016). Preparation and characterization (Mechanical and water absorption properties) of CMC/PVA/clay nanocomposite films. Iran. J. Chem. Chem. Eng..

[31] Golizadeh M., Karimi A., Gandomi-Ravandi S., Vossoughi M., Khafaji M., Joghataei M.T., Faghihi F. (2019). Evaluation of cellular attachment and proliferation on different surface charged functional cellulose electrospun nanofibers. Carbohydr. Polym..

[32] Pouranvari S., Ebrahimi F., Javadi G., Maddah B. (2016). Chemical cross-linking of chitosan/polyvinyl alcohol electrospun nanofibers. Mater. Tehnol..

[33] Park Y., You M., Shin J., Ha S., Kim D., Heo M.H., Nah J., Kim Y.A., Seol J.H. (2019). Thermal conductivity enhancement in electrospun poly(vinyl alcohol) and poly(vinyl alcohol)/cellulose nanocrystal composite nanofibers. Sci. Rep..

[34] Khan M.Q., Kharaghani D., Nishat N., Ishikawa T., Ullah S., Lee H., Khatri Z., Kim I.S. (2018). The development of nanofiber tubes based on nanocomposites of polyvinylpyrrolidone incorporated gold nanoparticles as scaffolds for neuroscience application in axons. Text. Res. J..

[35] Ignatova M., Manolova N., Rashkov I. (2007). Novel antibacterial fibers of quaternized chitosan and poly(vinyl pyrrolidone) prepared by electrospinning. Eur. Polym. J..

[36] Abdelrazek E.M., Abdelghany A.M., Tarabiah A.E., Zidan H.M. (2019). AC conductivity and dielectric characteristics of PVA/PVP nanocomposite filled with MWCNTs. J. Mater. Sci. Mater. Electron..

[37] Kaur R., Tripathi S.K. (2015). Study of conductivity switching mechanism of CdSe/PVP nanocomposite for memory device application. Microelectron. Eng..

[38] Safo I.A., Werheid M., Dosche C., Oezaslan M. (2019). The role of polyvinylpyrrolidone (PVP) as a capping and structure-directing agent in the formation of Pt nanocubes. Nanoscale Adv..

[39] Irfan M.S., Gill Y.Q., Ullah S., Naeem M.T., Saeed F., Hashmi M. (2019). Polyaniline-NBR blends by in situ polymerization: Application as stretchable strain sensors. Smart Mater. Struct..

